# Detection of Arboviruses and Other Micro-Organisms in Experimentally Infected Mosquitoes Using Massively Parallel Sequencing

**DOI:** 10.1371/journal.pone.0058026

**Published:** 2013-02-27

**Authors:** Sonja Hall-Mendelin, Richard Allcock, Nina Kresoje, Andrew F. van den Hurk, David Warrilow

**Affiliations:** 1 Public Health Virology Laboratory, Queensland Health Forensic and Scientific Services, Archerfield, Queensland, Australia; 2 LotteryWest State Biomedical Facility – Genomics, School of Pathology and Laboratory Medicine, University of Western Australia, Perth, Western Australia, Australia; 3 Department of Clinical Immunology, Pathwest Laboratory Medicine WA, Royal Perth Hospital, Perth, Western Australia, Australia; Blood Systems Research Institute, United States of America

## Abstract

Human disease incidence attributed to arbovirus infection is increasing throughout the world, with effective control interventions limited by issues of sustainability, insecticide resistance and the lack of effective vaccines. Several promising control strategies are currently under development, such as the release of mosquitoes trans-infected with virus-blocking *Wolbachia* bacteria. Implementation of any control program is dependent on effective virus surveillance and a thorough understanding of virus-vector interactions. Massively parallel sequencing has enormous potential for providing comprehensive genomic information that can be used to assess many aspects of arbovirus ecology, as well as to evaluate novel control strategies. To demonstrate proof-of-principle, we analyzed *Aedes aegypti* or *Aedes albopictus* experimentally infected with dengue, yellow fever or chikungunya viruses. Random amplification was used to prepare sufficient template for sequencing on the Personal Genome Machine. Viral sequences were present in all infected mosquitoes. In addition, in most cases, we were also able to identify the mosquito species and mosquito micro-organisms, including the bacterial endosymbiont *Wolbachia*. Importantly, naturally occurring *Wolbachia* strains could be differentiated from strains that had been trans-infected into the mosquito. The method allowed us to assemble near full-length viral genomes and detect other micro-organisms without prior sequence knowledge, in a single reaction. This is a step toward the application of massively parallel sequencing as an arbovirus surveillance tool. It has the potential to provide insight into virus transmission dynamics, and has applicability to the post-release monitoring of *Wolbachia* in mosquito populations.

## Introduction

The domesticated mosquito, *Aedes aegypti*, is the primary vector of devastating diseases such as dengue (DENV), yellow fever (YFV) and chikungunya (CHIKV) viruses. These viruses can cause large epidemics [1], [2], [3] and, as they are arthropod-borne, are commonly referred to as arboviruses. Another peri-domestic mosquito, *Aedes albopictus*, has recently emerged as a major vector of CHIKV and is a secondary vector of dengue or primary vector during outbreaks where *Ae. aegypti* populations are low or absent. Although a highly efficacious vaccine has been administered against YFV for over 80 years, declining immunization practices in conjunction with growing urbanization have led to rising numbers of cases. Vaccines against the DENVs and CHIKV are still under development. Thus, control of *Ae. aegypti* and *Ae. albopictus* populations via elimination of larval habitats and application of insecticides remains the primary approach for minimizing virus transmission [4]. Unfortunately, effective mosquito control programs are difficult to sustain and alternative strategies are desperately required.

A novel biological control approach involving the release of an experimentally-generated *Ae. aegypti* line carrying maternally-transmitted bacteria (*Wolbachia*) is being developed [5]. Mosquitoes carrying this strain exhibit significantly reduced replication of DENVs, CHIKV and YFV [6], [7], [8]. In addition, *Wolbachia* confers a biological advantage referred to as cytoplasmic incompatibility, which facilitates its drive into natural mosquito populations [9]. Due to this advantage, controlled release of *Wolbachia*-infected mosquitoes should theoretically result in fixation of a virus-resistant population. Unlike *Ae. aegypti*, *Ae. albopictus* harbors two naturally occurring strains of *Wolbachia*, *w*AlbA and *w*AlbB. However, these strains have not been linked to overt inhibition of arbovirus replication. Thus, a virus-blocking strain of *Wolbachia*, *w*Mel was recently trans-infected into *Ae. albopictus* resulting in reduced DENV-2 replication [10].

*Wolbachia*-infected *Ae. aegyp*ti mosquitoes were released in northern Queensland, Australia, in 2011 and reached rapid fixation [11]. There are plans to release trans-infected *Ae. aegyp*ti mosquitoes in Asia and other endemic countries [5]. It will be essential to monitor mosquito populations to determine whether the *Wolbachia* infection in natural populations is maintained through multiple generations post-release. Commensurate with this, it will be necessary to analyze mosquitoes for arbovirus infection to determine whether the presence of *Wolbachia*-infected populations is suppressing the arbovirus transmission cycle. Traditionally, detection of arboviruses in mosquito populations is conducted by isolating viable virus or detecting viral RNA in field collected mosquitoes [12]. Thus, mosquitoes being collected for *Wolbachia* detection can be screened for arboviruses, as well as for genetic structure of the mosquito population at release locations [13]. In the case of blood engorged specimens, identification of the vertebrate origin of the blood meal is also possible [14].

There is a need to develop new molecular-based approaches to examine the interrelationships between vectors, endosymbiotic bacteria and arboviruses in mosquito control programs, such as the release of *Wolbachia*-infected mosquitoes and in arbovirus surveillance programs more generally. Massively parallel sequencing (MPS) technologies have enormous potential application for arbovirus surveillance [15], [16], [17]. Due to the ability to generate massive amounts of sequence data in parallel, a shotgun approach to sampling can be taken, which presents a number of advantages. In terms of virus detection and identification, data can be obtained directly from the sample, bypassing a requirement to obtain a culture isolate, and enabling the detection of viruses that are not amenable to culture. Second, whole viral genome information may be obtained, maximizing the genetic information from the virus. Third, these methods also present the opportunity to obtain genetic information from the vector such as insect and microbial ribosomal RNA sequence data. This aspect would have particular application in monitoring the *Wolbachia* distribution in mosquito populations, post release. Obtaining genetic information on the mosquito may provide insights into genetic structure, gene flow and even identification of damaged specimens or cryptic species.

There are few examples of application of MPS to detection of virus in mosquitoes. These include detection of densovirus in wild caught *Culex pipiens molestus* [18] and DENV-1 in laboratory-infected *Ae. aegypti* [19], and a broad survey of wild caught mosquitoes [20]. In this work, we have used experimental infection of the two most important arbovirus mosquito vectors, *Ae. aegypti* and *Ae. albopictus*, to determine the applicability of MPS for both virus detection and analysis of host genetic information. Not only were we able to readily detect DENV-3, YFV and CHIKV sequences, but we were able to simultaneously obtain sequence information on associated micro-organisms, including *Wolbachia*, and on the mosquito host itself. We found MPS to be an effective tool when using one of the less expensive platforms, such as the Personal Genome Machine (PGM, Life Technologies). This emergent technology has the potential to provide important information that can be applied to control strategies aimed at minimizing the burden of arboviral disease.

## Materials and Methods

### Virus strains

Chikungunya virus was isolated in Australia from a traveller from Mauritius in March 2006. DENV-3 was isolated from a patient during the 2008 outbreak in Cairns in North Queensland, Australia. Cenetrop 28 (OBS 7549), a yellow fever virus strain from South America, was isolated from a patient in 1999.

### Mosquitoes

*Ae. aegypti* trans-infected with *Wolbachia* were obtained from a line generated by micro-injection of the *w*Mel strain which was originally derived from *Drosophila melanogaster* [7]. The *Ae. albopictus* were obtained from a colony established from material collected from Yorke Island in the Torres Strait, northern Australia.

### Laboratory infection of mosquitoes with CHIKV, DENV-3 and YFV

All mosquito infections were performed in an approved Biological Safety Level 3 insectary. *Ae. albopictus* were exposed to DENV-3 and CHIKV via an infectious blood meal housed in a membrane feeding apparatus. The blood meal was prepared by adding stock virus to washed defibrinated sheep blood sweetened with 1% sucrose to produce final titres of 10^6.1^ and 10^5.7^ tissue culture infectious dose (TCID)_50_/ml of DENV-3 and CHIKV, respectively. *Ae. aegypti* were exposed to YFV via intrathoracic inoculation with 0.5 μl of a 10^4^ TCID_50_/mL dilution of stock virus. Post exposure, mosquitoes were maintained on 10% sucrose at 28°C, 75% RH and 12∶12 L:D (light:dark). After 14 d, mosquitoes were killed and stored at −80°C. Virus infection was confirmed by TaqMan-based quantitative PCR assay.

### Amplification of mosquito nucleic acids

Infected mosquitoes, and an uninfected control, were homogenized in Opti-MEM with 3% fetal bovine serum for 2 min using a TissueLyser II automated shaker in a capped 1.5 ml tube containing a metal bead (Qiagen). Nucleic acids were extracted from the homogenate using a QIAamp viral RNA extraction kit (Qiagen) following the manufacturer's recommendations with the exception that carrier RNA was omitted from the AVL buffer. One of two methods was used for the random amplification of mosquito RNA. The first method was based on the phage-derived Φ29 DNA polymerase. Firstly, RNA (9 μl of extract) was reverse transcribed using Multiscribe (Life Technologies) in a 20 μl reaction containing the supplied reaction buffer and 50 pmole of random hexamers. The mixture was incubated at 25°C for 5 min then 50°C for 1 h. The resulting cDNA was used as a template for GenomiPhi (GP; GE Healthcare) amplification. Briefly, the cDNA was denatured by heating at 95°C for 3 min in the supplied sample buffer containing random hexamers, then cooled on ice. The denatured cDNA was then mixed with the supplied reaction buffer containing the DNA polymerase and nucleotides, and the reaction was incubated at 30°C for 2.5 hours before heat inactivation of the enzyme at 65°C for 10 min. The second method was a modification of a previously described sequence-independent amplification [21]. RNA (5 μl of extract) was used as a template for reverse transcription with a modified random primer (K-15N 5′-GACCATCTAGCGACCTCCACNNNNNNNNNNNNNNN-3′) and second strand DNA synthesis using the SuperScript™ III One-Step RT-PCR System with Platinum® Taq High Fidelity mix (Invitrogen) with cycling [25°C for 5 min; 37°C for 30 min; 95°C for 2 min]_1_ [95°C, 30 sec; 25°C, 30 sec; 72°C, 2 min]_5_. The product of this reaction (2 μl) was used as a template for amplification in a mix containing an amplification primer (Primer K 5′-GACCATCTAGCGACCTCCAC-3′) and using AmpliTaq Gold thermostable DNA polymerase with cycling: [95°C, 12 min]_1_ [95°C, 30 sec; 59°C, 30 sec; 72°C, 2 min]_50_ [72°C, 7 min]_1_ [15°C]_hold_.

### Library preparation

Dried samples were resuspended in 50 μl of water and sheared to approximately 200 bp fragments using an S2 sonicator (Covaris, Inc. MA, USA). Sequencing libraries were prepared with an Ion Xpress Plus Fragment Library Kit (Life Technologies, NY, USA). Individual samples were barcoded prior to sequencing. Fragments of approximately 330 bp were then excised from an agarose gel to ensure a high proportion of full-length sequencing templates and the libraries were quantified using a High Sensitivity DNA chip on a Bioanalyzer 2100 (Agilent Technologies, CA, USA).

### Template preparation and sequencing

Barcoded samples were pooled in equimolar ratios to a total concentration of 9 pM in low TE buffer. Template preparation and enrichment was performed using an Ion OneTouch Template 200 Kit (Life Technologies, NY, USA) on a OneTouch and OneTouch ES (Life Technologies, NY, USA). Sequencing was performed using an Ion PGM 200 Sequencing Kit on “316” sequencing chips for a total of 520 nucleotide flows, yielding average read lengths of 220–230 bp. Five or six samples were pooled on a single chip, generally yielding >450,000 reads per sample.

### Bioinformatics

Primary analysis (ie. base-calling and barcode de-convolution) was performed using Torrent Suite 2.2. Initially, reads were trimmed of adaptor sequences and filtered to remove polyclonal and low quality reads. They were then trimmed to remove poor quality bases at the 3′ end of long reads. A FASTQ file containing the output from PGM sequencing was imported into GeneiousPro software [22]. Sequence reads were assembled using default parameters against a relevant reference sequence (see Table 1). For database searching, individual reads were matched against either the NCBI viral reference sequence database [23] or the SILVA small and large ribosomal subunit databases [24] using standalone BLASTn with default parameters. The blast output file was imported into the program MEGAN (for MEtaGenomic ANalyser) [25], which assigns matches using a lowest common ancestor (LCA) approach on the basis of NCBI taxonomy, as a graphic output. The LCA parameters were set to a Minimum Score of 100, a Top Percentage of 10, and Minimum Support of 10. Multiple sequence alignment was performed with the Geneious Alignment feature of GeneiousPro, and the output file used to calculate a distance matrix using a Jukes Cantor model with bootstrapping (1000 replicates) and a phylogenetic tree using neighbour joining with MEGA5 [26].

**Table 1:** Detection of virus in experimentally-infected mosquitoes by MPS.

*Mosquito number*	*Species*	*Method*	*Virus^1^*	*C_t_^2^*	*Total reads*	*Reads matched to virus (%)^3^*	*Genome*	
							*Coverage (%)*	*Max. depth*
1	*Ae. albopictus*	SIA	DENV-3	22.8	696,482	36,290 (5%)	97	1,762
2	"	"	DENV-3	22.9	439,421	894 (0.2%)	63	72
3	"	"	CHIKV	15.6	530,479	188,810 (35%)	45	11,079
4	"	"	CHIKV	16.9	419,562	730 (0.2%)	33	51
5	*Ae. aegypti*	"	YFV	18.1	448,194	5,342 (1.2%)	55	859
"	"	"	Densovirus	na4	"	23 (0.005%)	na	na
"	"	GP	YFV	17.1	365,695	4,408 (1.2%)	85	317
"	"	"	Inovirus	na	"	3035 (0.8%)	na	na

1. Reference sequences (NCBI accession number): DENV-3, NC_001475.2; CHIKV, DQ443544.2; YFV, NC_002031.1.

2. Detection by Taqman assay – threshold value (C_t_).

3. For DENV-3, CHIKV and YFV, values were the number of reads matched using the reference assembly feature of GeneiousPro. For densovirus and inovirus, values were the number of reads matched by standalone BLASTn followed by MEGAN.

4. Not applicable.

## Results and Discussion

### Detection of viruses in mosquitoes

To determine the feasibility of using MPS for detecting and genotyping arboviruses, two DENV-3 infected mosquitoes, two CHIKV infected mosquitoes, and a single YFV infected mosquito were analysed. Nucleic acid extracts prepared from homogenates of individual mosquitoes were randomly amplified using either a Φ29 polymerase based reaction (GenomiPhi, GE Healthcare), or sequence-independent amplification (SIA). In the case of the SIA method, a primer with 15 random nucleotides at the 3′ end was used for reverse transcription. Use of this primer avoided the recognized mis-priming associated with primers with shorter random sequences [27]. In optimization experiments we also found more even coverage using this primer (data not shown). Amplified reaction products were barcoded and used as template for sequencing on a PGM using a 316 chip.

**Figure 1:**
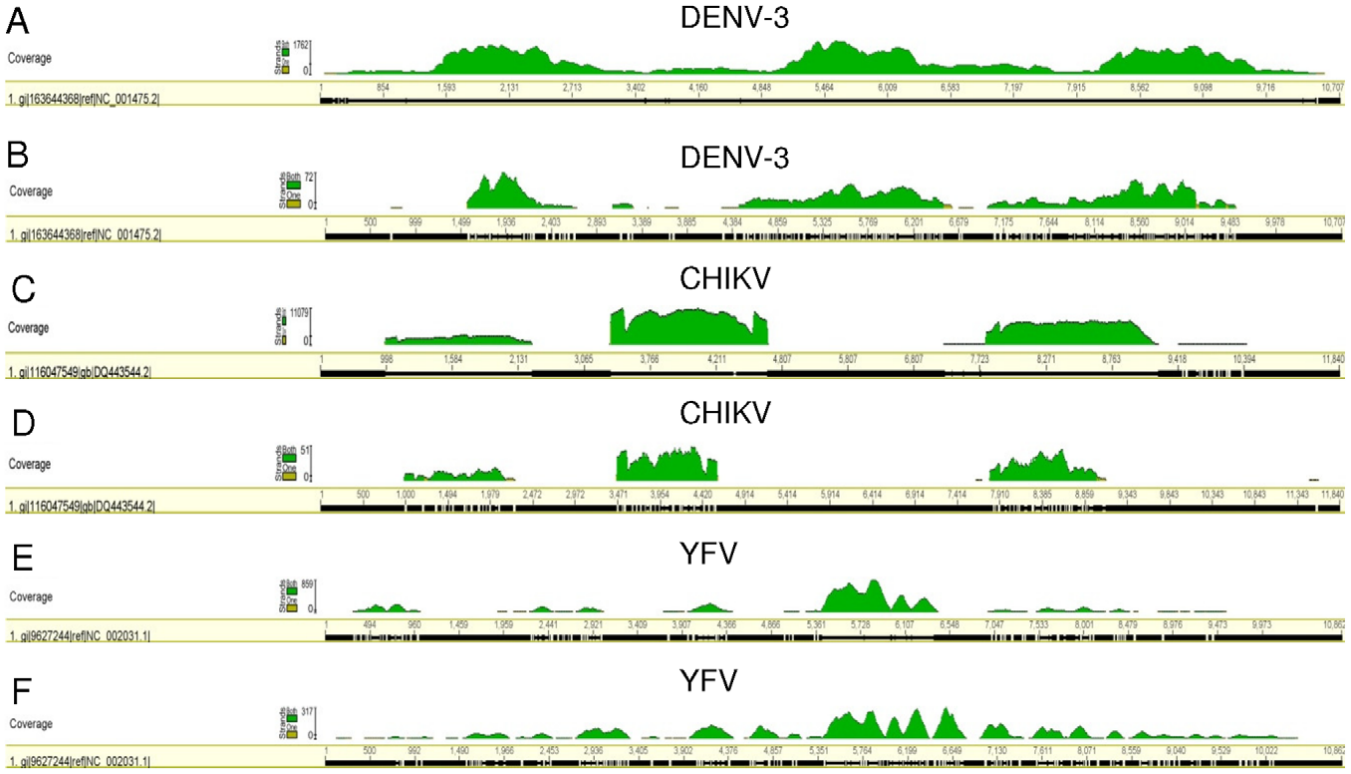
Comparison of sequence coverage and depth of the virus genomes. Products were generated using sequence-independent amplification from mosquitoes 1 and 2, which were infected with DENV-3 (A and B, respectively), mosquitoes 2 and 3, which were infected with CHIKV (C and D, respectively), and mosquito 5, which was infected with YFV and amplified either using sequence-independent amplification (E) or GenomiPhi amplification (F). These products were sequenced and assembled to a reference sequence as shown below the coverage diagram for each sample (DENV-3, NC_001475.2; CHIKV, DQ443544.2; YFV, NC_002031.1). The genome coverage (x-axis) and depth of coverage (y-axis) is shown.

Virus sequences were detected in laboratory-infected mosquitoes, and were assembled to a viral reference sequence. The distribution of coverage of the assembled sequences is shown in Figure 1 and the results are summarized in Table 1, with genome coverage and maximum depth as shown. No reads corresponding to virus sequences used in this study were detected in an uninfected *Ae. albopictus* control. Genome coverage varied between samples and, as might be expected, was generally improved with a relatively larger number of reads matching the infecting virus genome. One exception to this was mosquito 3 (Fig. 1C) which had 188,810 matches to the CHIKV genome with relatively low coverage (45%) but excellent maximum depth (11,079). This may have been due to some degree of complementarity between the amplification primer used in the SIA method and the viral genome resulting in biased amplification of a proportion of the genome. This conclusion was supported by the coverage of mosquito 4 which had a very similar distribution of reads (Fig. 1D).

Both of the amplification methods, GP and SIA used to amplify nucleic acids from the mosquito infected with YFV (mosquito 5) resulted in the successful detection of the virus RNA genome. Both amplification methods were similarly sensitive in being able to detect the virus sequences, however, the GP method provided better genome coverage. In comparison, the SIA method was better able to detect the mosquito ribosomal RNAs, whilst both were able to detect bacterial ribosomal RNAs. The proportion of reads that matched to the inoculated virus varied between duplicate DENV-3 and CHIKV samples when SIA was used. However, quantitative RT-PCR indicated similar amounts of virus in the mosquitoes (Table 1). This observation cannot currently be explained but may be related to the stochastic nature of the SIA or, alternatively, the PGM sequencing method.

In addition to those arboviruses experimentally inoculated into mosquitoes, two additional viruses were fortuitously detected in mosquito 5 (*Ae. aegypti*) using a standalone search of the NCBI viral reference database using BLASTn software followed by MEGAN analysis of the output (Table 1). MEGAN assigns groups taxonomically by lowest common ancestor (LCA) [28]. In the sample amplified by SIA, 23 reads were matched to densovirus, a virus previously reported in *Ae. albopictus* and *Ae. aegypti* [29], [30], and *Culex pipiens molestus* and *Culex pipiens pallens* mosquitoes [18], [31], as well as during surveillance of wild mosquitoes [20], and is a known contaminant in a number of mosquito cell lines [32]. In the sample amplified using the GenomiPhi method from the same mosquito, 3035 reads matching an inovirus were detected. Inoviruses are bacteriophage often associated with enteric bacterial hosts [33]. The phage sequences detected may be from a bacteriophage infecting a bacterium in the gut of the mosquito.

### Detection of mosquito RNA

Individual sequence reads were also matched against the SILVA small and large ribosomal database using standalone BLAST and MEGAN analysis as above [28]. All of the *Ae. albopictus* samples (4 infected) were successfully typed as such (Table 2). However in some samples, a small proportion reads were incorrectly assigned by MEGAN at the chosen parameter settings. For example, in one of the *Ae. albopictus* samples some of the sequence reads were matched to *Ae. aegypti*. However, the vast majority of reads were correctly assigned to *Ae. albopictus* in this case and so the sample was correctly typed on this basis. The lowest taxonomic level to which the *Ae. aegypti* sample could be assigned was order (ie. Diptera).

**Table 2:** Mosquito and micro-organism ribosomal RNA analysis.

*Mosquito number*	*Species*	*Method*	*LCA assignment (level)^1^*		*Micro-organism*	*LCA assignment (level)^1^*	
			*Small rRNA*	*Large rRNA*		*Small rRNA*	*Large rRNA*
1	*Ae. albopictus*	SIA	119 (S)	26,680 (S)	*Wolbachia*	26 (G)	496 (G)
					*Bukholderia*	-	19 (G)
2	"	"	31 (S)	4,306 (S)	*Wolbachia*	-	26 (G)
3	"	"	538 (S)	17,276 (S)	*Wolbachia*	359 (G)	4,926 (G)
4	"	"	699 (S)	48,824 (S)	*Wolbachia*	1,683 (G)	18,907 (G)
5	*Ae. aegypti^3^*	"	1,169 (O)	1,163 (O)	*Wolbachia*	1,534 (G)	159 (S)2
					*Asaia*	11 (G)	-
					*Enterobacteriaceae*	10 (F)	-
					*Flavobacteriaceae*	-	173 (F)
					*Trichocomaceae-Leotiomyceta*	12 (F)	15 (T)
5	"	GP	-	11 (T)4	*Cyanobacteria*	4,993 (P)	-
					*Pseudomonas*	1,777 (G)	-
					*Mycoplasma*	-	11 (G)

1. The number of reads assigned by MEGAN to the LCA taxonomic level indicated: species (S), genus (G), family (F), order (O), phylum (P), or rankless taxon (T).

2. *Wolbachia* endosymbiont of *D. melanogaster*.

3. Trans-infected with *Wolbachia*.

4. Taxon without rank, *Coelomata*.

### Detection of mosquito micro-organisms

Whilst our primary interest was the development of a method to ultimately detect infected mosquitoes for arbovirus surveillance, micro-organisms associated with mosquitos were fortuitously detected by analysis of ribosomal RNA species (Table 2). Of particular interest was the detection of *Wolbachia* sequences in all of the infected mosquito samples. This bacterium occurs naturally in *Ae. albopictus* as an obligate intra-cellular parasite [34], [35], [36], but in the case of the *Ae. aegypti* mosquito that had been trans-infected with the *w*Mel strain of *Wolbachia* [7]. Interestingly, the classification by LCA of *Wolbachia* sequences in the sample derived from *Ae. albopictus* was at the genus level whereas a significant proportion of the sequences derived from the *Ae. aegypti* were classified to the species level as “*Wolbachia* endosymiont of *Drosophila melanogaster*” (Table 2). This is consistent with *Wolbachia* from *D. melanogaster* being the source of inoculum for trans-infection [7], and suggests that the method was able to differentiate between natural and trans-infected sources of *Wolbachia*.

**Figure 2:**
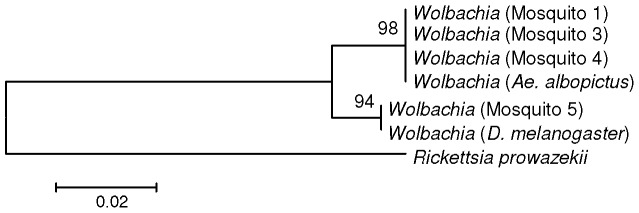
Relationship of *Wolbachia* derived from *Ae. albopictus* and *Ae. aegypti* mosquitoes based on 16S ribosomal RNA sequence. Ribosomal RNA sequences were assembled using a reference sequence, and a multiple alignment file was constructed. This was used to calculate a distance matrix and phylogenetic tree using the neighbour joining method. For comparison, *Wolbachia* 16S ribosomal RNA from field-caught *Ae. albopictus* (Accession number X61767.1) and *D. melanogaster* (Accession number AE017196.1) were included, and 16S ribosomal RNA from *Rickettsia prowazekii* (Accession number NC_017560.1) was used as an out-group. The numbers shown are the bootstrapping values for 1000 replicates.

To further explore this result, 16S ribosomal RNA sequences from the relevant samples were extracted and assembled, and a phylogenetic analysis performed to compare *Wolbachia* sequences from the *Ae. albopictus* and *Ae. aegypti* samples with *Wolbachia* sequences from field caught *Ae. albopictus* and *D. melanogaster* (Fig. 2). Both trees showed grouping of the *Ae. albopictus* samples with wild-caught mosquitoes, whilst the sequences derived from trans-infected *Ae. aegypti* grouped with *Wolbachia* from *D. melanogaster*. Hence, the phylogenetic analysis confirmed the method was able to differentiate between natural and trans-infected *Wolbachia* in mosquitoes.

Sequences which matched with a member of the genus *Burkholderia*, which has been reported previously in *Anopheles gambiae* [37], were also detected. These were the only bacterial sequences detected other than *Wolbachia* in the four *Ae. albopictus* samples. This contrasted with the *Ae. aegypti* sample where a number of other bacterial taxonomic groups were identified (Table 2). These included the genus *Asaia*, an alphaproteobacteria which has been previously identified in both laboratory and field strains of *Ae. aegypti* [19], [35], [38], [39]. This bacterial group has also been detected in *Ae. albopictus*, *An. gambiae* and *An. stephensi* [40]. In the latter mosquito species its symbiosis is important for larval development [41]. Sequences that could be classified with the *Enterobacteriaceae* were also detected, which is consistent with previous detection in field caught *Ae. aegypti* and *Ae albopictus* [35]. Both *Asaia* and *Enterobacter* species have been detected in *Ae. aegypti* eggs indicating the potential for transovarial transmission [38].

Sequences from other bacteria included the cyanobacteria, *Flavobacteriaceae*, and *Pseudomonas*. Cyanobacteria have been detected in *An. gambiae* primarily during larval and pupal stages [42], [43], [44], [45], [46] and are thought to be a food source during mosquito larvae development [47], [48], [49], [50], [51]. The large number of hits suggests it may be a significant part of the microflora in this instance. *Flavobacteriaceae* and *Pseudomonas* have been detected previously in *Ae. aegypti* [35], [39], *Aedes triseriatus*, *Culex pipiens*, *Psorophora columbiae*, *Culex quinquefasciatus* and anophelines [52], [53], [54], [55], [56]. Mycoplasma sequences, which have not previously been reported in any mosquito species, but have been reported in ticks [57], were also detected. Non-bacterial micro-organism sequences detected were the fungi (*Trichocomaceae* family), which has previously been isolated from *Ae. aegypti* and other mosquito species [19], [58].

In these experiments, we have demonstrated the feasibility of the use of MPS for arbovirus surveillance. The method is capable of detecting arboviruses of medical importance, as well as known bacterial and other micro-organisms in a sequence-independent manner. The detection of sequences from a small number of micro-organisms associated with mosquitoes was fortuitous, but would require deep-sequencing of PCR products generated using specific primer sets to conserved ribosomal RNA sequences to be a thorough analysis. However, it did demonstrate the ability of MPS of randomly amplified material to detect a large number of diverse microbiological targets. It is a significant advancement in the development of a workable surveillance method. The use of laboratory-infected mosquitoes enabled appropriate software parameters for future field work to be established. Future trials will determine the sensitivity and reliability of the method in the field. Currently, this technology is not cost competitive in comparison with PCR-based screening methods. However, MPS technology is currently in its infancy, and as costs continue to decline it will increasingly become a valuable tool for the surveillance of known and novel arboviruses, and monitoring *Wolbachia* release programs for dengue control.
